# Supplementary material for: Beneficial effects of upgrading to His-Purkinje system pacing in patients with pacing-induced cardiomyopathy: a systematic review and meta-analysis
Source: PeerJ. 2023 Oct 11;11:e16268. doi: 10.7717/peerj.16268 (PMC10576494; doi:10.7717/peerj.16268)
Supplement: Supplemental Information 2 [file peerj-11-16268-s002.docx]

## Systematic Review and/or Meta-Analysis Rationale

For systematic reviews / meta-analyses, authors need to provide the following information:

1. The rationale for conducting the systematic review / meta-analysis;

Responds:

We would like to thanks for your comments. Right ventricular pacing induced cardiomyopathy (PICM) is a common cause of cardiac dysfunction with a high incidence. Although cardiac resynchronization therapy (CRT) can improve PICM, it is not always feasible, and the proportion of patients without CRT response remains high. His-purkinje system pacing (hsp) is regarded as the most physiologic cardiac pacing mode, promising to improve cardiac dysfunction in patients with right ventricular pace-induced cardiomyopathy (PICM). The value of HPSP in PICM is still mixed. Therefore, we conducted this systematic review and meta-analysis to determine the effectiveness of HPSP upgrades in patients with PICM.

1. The contribution that it makes to knowledge in light of previously published related reports, including other meta-analyses and systematic reviews.

Responds:

Thank you very much for the comments. We included the most recent relevant studies to date for this systematic review and meta-analysis. Besides, we firstly compare the safety and efficacy of HBP and LBBP in PICM patients.
