# Supplementary figures and images for: Beneficial effects of upgrading to His-Purkinje system pacing in patients with pacing-induced cardiomyopathy: a systematic review and meta-analysis
Source: PeerJ. 2023 Oct 11;11:e16268. doi: 10.7717/peerj.16268 (PMC10576494; doi:10.7717/peerj.16268)

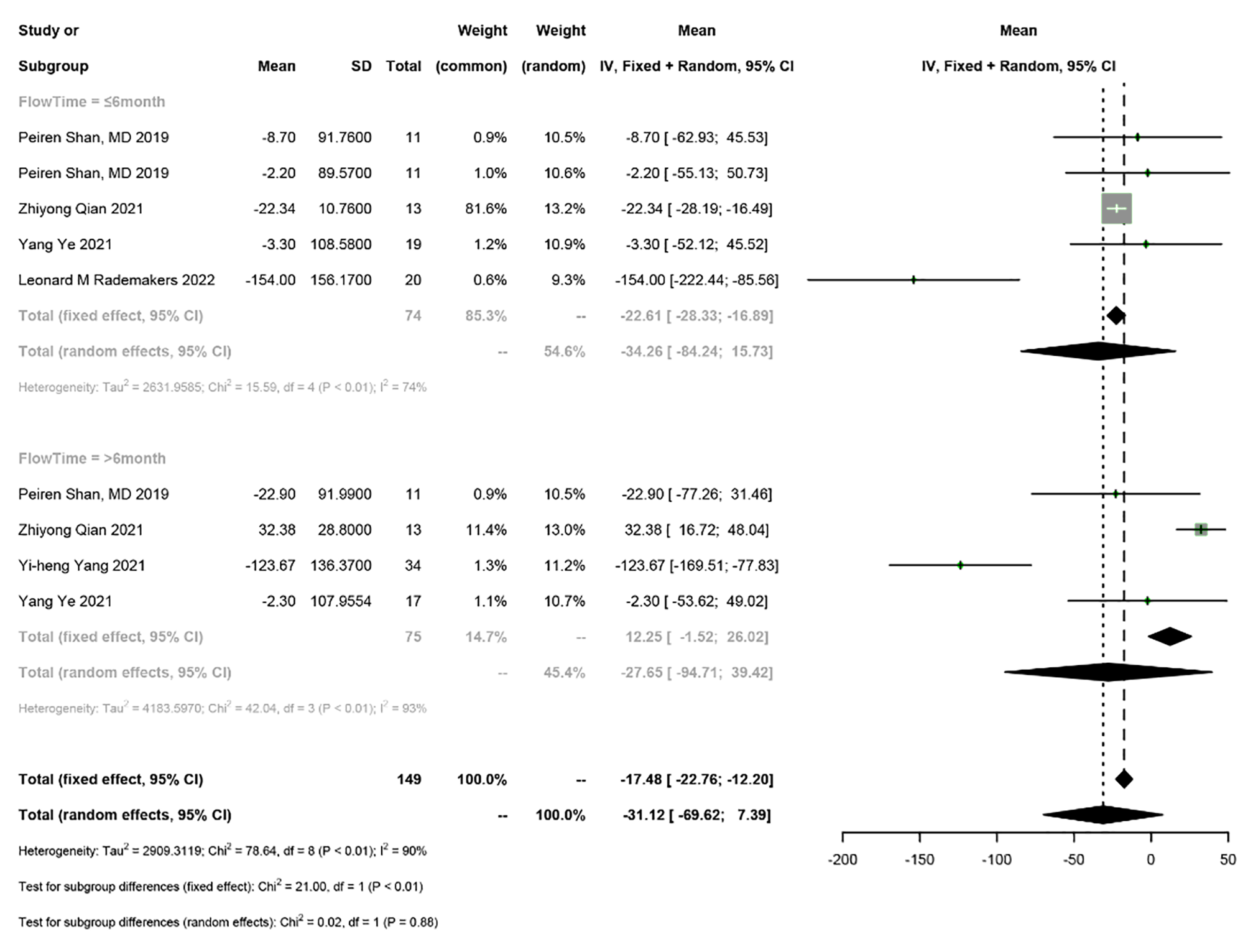

Supplement: Supplemental Information 3 [file peerj-11-16268-s003.png]
